# Coil Positioning for Wireless Power Transfer System of Automatic Guided Vehicle Based on Magnetic Sensing

**DOI:** 10.3390/s20185304

**Published:** 2020-09-16

**Authors:** Ce Liang, Yanchi Zhang, Zhonggang Li, Feng Yuan, Guang Yang, Kai Song

**Affiliations:** 1 School of Instrument Science and Engineering, Harbin Institute of Technology, Harbin 150006, China; 18741370524@163.com (C.L.); 1170100409@stu.hit.edu.cn (Y.Z.); zhonggangli@hit.edu.cn (Z.L.); yuanf@hit.edu.cn (F.Y.); 2 School of Electrical Engineering & Automation, Harbin Institute of Technology, Harbin 150006, China; guangyang@hit.edu.cn

**Keywords:** wireless power transfer (WPT), coil positioning, Hall sensor array, positioning algorithm

## Abstract

As an auxiliary function of the wireless power transfer (WPT) system, coil positioning can solve the power and efficiency degradation during power transmission caused by misalignment of the magnetic coupler. In this paper, a Hall sensor array is used to measure the change of magnetic flux density. By comparing the multisensor data fusion results with the preset data obtained from the coil alignment, the real-time accurate positioning of the receiving coil can be realized. Firstly, the positioning model of the receiving coil is built and the variation of magnetic flux density with the coil misalignment is analyzed. Secondly, the arrangement of the Planar 8-direction symmetric sensor array and the positioning algorithm based on data fusion of magnetic flux density variations are proposed. In order to avoid coil positioning misalignment caused by the unstable magnetic field distribution which is actually affected by the change of mutual inductance during automatic guided vehicle (AGV) alignment, the constant current strategy of primary and secondary sides is proposed. Finally, the coil positioning experimental platform is built. The experimental results show that the coil positioning method proposed in this paper has high accuracy, and the positioning error is within 4 cm.

## 1. Introduction

Compared with wired charging, wireless power transfer (WPT) is widely used in electric vehicles, automatic guided vehicles (AGVs), implant applications and other fields for its convenience, safety and reliability [1,2,3,4,5,6,7]. However, new problems have arisen. The misalignment of the transmitting coil and receiving coil will lead to a decrease in WPT efficiency and the high voltage stress caused by reactive power. Serious misalignment can result in system failure [8,9]. Detection of coil alignment before the charging process can improve the performance of the WPT system [10].

**Figure 1:**
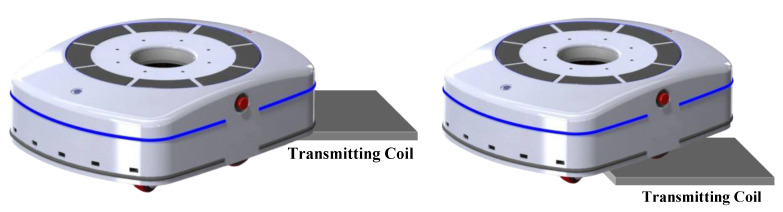
Coil misalignment diagram.

Since the transmitter and the receiver of a WPT system are noncontact, coil misalignment is a common phenomenon, as shown in Figure 1. For the purpose of enhancing tolerance for misalignment, some methods have been proposed such as optimize the electric coupler [11,12], improve circuit topology [13,14,15], frequency tracking [16] and so on. The above schemes can extraordinarily expand the charging range of the WPT system. Nevertheless, these methods would be invalid when the coupling effect between the transmitter and receiver is fairly weak. Therefore, it is necessary to study the coil positioning method to eliminate the misalignment and ensure the efficient operation of a wireless power transfer (WPT) system. As shown in Table 1, various positioning methods based on global positioning system (GPS) [17,18], camera [19], radio frequency identification (RFID) [20,21] and ultrawide bandwidth (UWB) [22] have been proposed. The accuracy of the GPS positioning system is affected by the external environment, and its accuracy is relatively low. RFID positioning systems require multiple tags and card readers for better positioning accuracy. In addition, the installation of tags and readers is another disadvantage limiting their application. In camera positioning systems, the electronic tags are often obscured by dirt or snow under some adverse factors such as bad weather, which makes the positioning accuracy drop dramatically. The UWB positioning system improves the detection accuracy, albeit at a high cost.

Owing to the advantages of having a low cost and high precision, the positioning method based on magnetic sensing has become a research focus of the coil positioning method for WPT systems. In [23], a precise location detection method for WPT systems based on a planar compact magnetoresistance sensor array was presented. The magnetic field change caused by coil misalignment is directly detected for positioning. The accuracy is 1 cm. However, both theoretical analysis and simulation are aimed at low-power human implantable devices with absolute insulation protection and are not suitable for AGV WPT systems exposed to the external environment. A positioning method for measuring magnetic flux density by laying multiple auxiliary coils at the receiver is proposed in [24]. When applied to the AGV WPT system, installing the auxiliary coil on the receiver cannot meet the lightweight requirements. Furthermore, when a metal foreign object appears at the transmitter, the positioning accuracy will be seriously affected. A dual-purpose coil installation for metal foreign object detection and coil position detection is proposed in [10]. The position of the receiving coil can be determined by measuring the change of the induced voltage of the detection coil. However, the position of the transmitting coil needs to be inferred from the expected results through simulation, depriving the coil positioning method of the real-time feature. It needs to be calculated in detail according to the parameters of AGV, which is quite complex to realize. A ferrite position identification (FPID) system is designed to provide accurate train location information, which is not affected by WPT electromagnetic interference [25]. This position detection method completes the positioning by detecting ferrite. However, this method fails in some applications of WPT systems without ferrite structure. In addition, in order to avoid the influence of WPT electromagnetic interference, the system is designed to be more complex. It is not universal to extend this method to the AGV WPT system.

In summary, there are some improvements in the coil positioning method based on the magnetic coupling of the AGV WPT system:

1. The coil positioning method has some drawbacks such as poor real-time performance and complex calculation process of positioning algorithm [10].
2. The effect of current change in primary and secondary sides caused by coil misalignment on magnetic field distribution is ignored. It is not strictly guaranteed that the current is constant and the detection accuracy is reduced [10,23,24].
3. The detection accuracy is limited by the application scenario and external factors, and the universality of the detection system is insufficient [23,24,25].

This paper presents a real-time and high-precision coil positioning method based on magnetic field detection and multisensor data fusion which is low-cost and widely applicable. Coil positioning is separated from WPT, which eliminates the influence of the magnetic field on the coil positioning process. Section 2 establishes the equivalent model structure of the receiving coil positioning system. Section 3 analyses the distribution of the magnetic field in precise alignment and offsets conditions among the transmitter and receiver from the perspective of electromagnetic field theory. The detection device is installed in the transmitter to realize the lightweight requirement of the receiver. The positioning algorithm of the receiving coil is proposed. In order to improve the accuracy and reliability of the detection, a constant current control strategy is proposed to strictly ensure the constant current throughout the detection process. Section 4 verifies the proposed coil positioning method by experiment.

## 2. Magnetic Coupler and Coil Positioning System Structure

**Figure 2:**
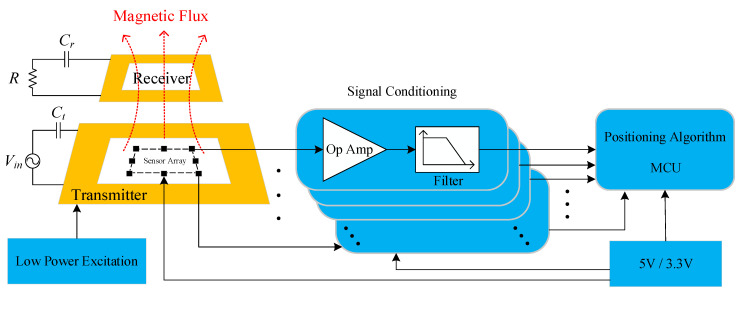
Structure of the coil positioning system.

A 1 kW magnetic coupler is designed for AGV. The specific parameters are shown in Table 2. The structure of the receiving coil positioning system is shown in Figure 2, including a low-power excitation source, a sensor array, the signal conditioning circuit and a microcontroller unit (MCU) for running the positioning algorithm. The process of coil positioning is carried out before charging and completed under low-power excitation, which means that the output power of the primary side is much less than the rated power of the system during the positioning process. It meets the detection range of the detection device and ensures the safety of the system. The linear Hall sensors are used to detect magnetic fields. The output signal of the linear Hall sensor is weak. In order to realize high-precision sampling, the signal conditioning circuits are designed, such as an amplifying circuit and a filtering circuit. Finally, the signal is connected to the MCU for analog–digital conversion, and the positioning algorithm is run to realize the precise positioning of the receiving coil.

*R* is the equivalent resistance of the load, *C_t_* and *C_r_* are the compensation capacitors at the transmitter and receiver, respectively, and *V_in_* is the transmitter input voltage.

## 3. Magnetic Field Distribution and Coil Positioning Algorithm

### 3.1. Spatial Magnetic Field Distribution Model

**Figure 3:**
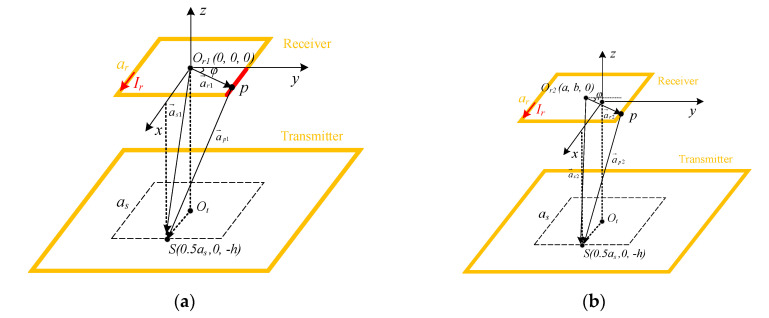
Magnetic flux density analysis: (**a**) without receiver misalignment; (**b**) with receiver misalignment.

As shown in Figure 3, a random sensing point S and a receiver relative position p are selected to illustrate how to calculate the magnetic flux density. Here, only the case that point p appears in the red line segment is discussed. Other cases can be derived from geometric relations and will not be repeated. In Figure 3, a_r_ and a_s_ are the side length of the square sensor array and the square receiver, respectively. O_r1_ and O_r2_ are the center coordinates of receiving coil alignment and misalignment, respectively. O_t_ is the center coordinate of the transmitting coil and h is the vertical distance between the transmitting coil and the receiver coil. Then, the magnetic flux density *B* in the z-axis component will be obtained to provide the receiver misalignment information in the xoy plane.

As shown in Figure 3a, when there is no misalignment, the vector $\vec{a_{p1}}$ which is the vector from a current element in the receiver to the sensing point can be represented by $\vec{a_{r1}}$ and $\vec{a_{s1}}$.

(1) $$\vec{a_{r1}}=\frac{a_{r}}{2}\tan\varphi \vec{e_{x}}+\frac{a_{r}}{2}\vec{e_{y}}$$

(2) $$\vec{a_{s1}}=\frac{a_{s}}{2}\vec{e_{x}}-h\vec{e_{z}}$$

(3) $$\vec{a_{p1}}=\vec{a_{s1}}-\vec{a_{r1}}=(\frac{a_{s}}{2}-\frac{a_{r}}{2}\tan\varphi )\vec{e_{x}}-\frac{a_{r}}{2}\vec{e_{y}}-h\vec{e_{z}}$$

where $\vec{e_{x}}$, $\vec{e_{y}}$ and $\vec{e_{z}}$ are the unit vectors in the x-, y- and z-directions, respectively, and φ is the angle between the vector $\vec{a_{r1}}$ and the y-axis.

According to the Biot–Savart law, the magnetic flux density *B* in the specified sensing point *S* (0.5*a_s_*, 0, −*h*) can be given by

(4) $$\vec{B}=\frac{\mu_{0}I_{r}}{4\pi }\int \frac{d\vec{l}\times \vec{a_{p1}}}{a_{p1}{}^{3}}$$

where $\mu_{0}$ is the vacuum permeability, *I_r_* is the exciting current, *a_p1_* is the norm of $\vec{a_{p1}}$ and $d\vec{l}$ is the wire element in the receiver.

(5) $$a_{p1}=\sqrt{\frac{a_{s}{}^{2}}{4}+h^{2}+\frac{a_{r}{}^{2}}{4}(1+\tan^{2}\varphi )-\frac{a_{r}a_{s}}{2}\tan\varphi }$$

(6) $$d\vec{l}=dx$$

Therefore, based on (4), (5) and (6), the z-direction component of the magnetic flux density can be calculated by

(7) $$\vec{B_{z1}}=\frac{\mu_{0}I_{r}}{4\pi }\int_{-\frac{a_{r}}{2}}^{\frac{a_{r}}{2}}\frac{(-1/2)a_{r}}{a_{p1}{}^{3/2}}dx$$

Then, when the misalignment occurs as shown in Figure 3b, the center of the receiver moves from *Q_r1_* (0, 0, 0) to *Q_r2_* (*a*, *b*, 0). The vector $\vec{a_{r2}}$, $\vec{a_{s2}}$ and $\vec{a_{p2}}$ can be obtained by

(8) $$\vec{a_{r2}}=\frac{a_{r}}{2}\tan\varphi \vec{e_{x}}+\frac{a_{r}}{2}\vec{e_{y}}$$

(9) $$\vec{a_{s2}}=(\frac{a_{s}}{2}\vec{e_{x}}-a)-b\vec{e_{y}}-h\vec{e_{z}}$$

(10) $$\vec{a_{p2}}=\vec{a_{s2}}-\vec{a_{r2}}=(\frac{a_{s}}{2}-a-\frac{a_{r}}{2}\tan\varphi )\vec{e_{x}}-(b+\frac{a_{r}}{2})\vec{e_{y}}-h\vec{e_{z}}$$

where *a* and *b* are the offset of the receiving coil in the x-axis and y-axis directions, respectively.

*a_p2_* is the length of the vector $\vec{a_{p2}}$ can be given by

(11) $$a_{p2}=\sqrt{{\left(\frac{a_{s}}{2}-a-\frac{a_{r}}{2}\tan\varphi \right)}^{2}+{\left(b+\frac{a_{r}}{2}\right)}^{2}+h^{2}}$$

Therefore, based on (4), (6) and (11), the z-direction component of the magnetic flux density can be calculated by

(12) $$\vec{B_{z2}}=\frac{\mu_{0}I_{r}}{4\pi }\int_{-\frac{a_{r}}{2}}^{\frac{a_{r}}{2}}\frac{-b-(1/2)a_{r}}{a_{p2}{}^{3/2}}dx$$

Equations (7) and (12) indicate that when the receiving coil approaches the sensing point, the magnetic flux density will increase with the decrease in the relative offset distance. The direction of the misalignment of the receiving coil can be determined by the maximum output value of the sensor. In addition, different offsets correspond to different magnetic flux density. Consequently, the relationship between different offset positions and magnetic flux density can be established to extract the location information of the AGV receiving coil. The receiving coil positioning algorithm will be described in detail below.

### 3.2. Spatial Magnetic Field Analysis and Positioning Algorithm of the Magnetic Coupler

**Figure 4:**
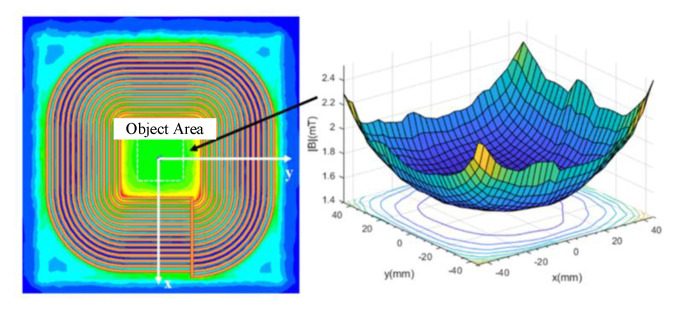
Distribution of magnetic flux density on the transmitting coil surface.

The coil positioning system is installed on the transmitter, considering the lightweight requirement of the receiver and the complexity of the system control. The research on the space magnetic field of the magnetic coupler focuses on the surface of the transmitter. The region with a positive center of 90 mm × 90 mm near the surface of the transmitter is selected as the object area, and the distribution of magnetic flux density is shown in Figure 4. The distribution of the magnetic field shows symmetric features on account that the coil structure is extremely symmetrical. As shown in Figure 4, the magnetic flux density near the Litz wire is high, while the magnetic flux density near the central region is relatively low. The magnetic field in space originates from the current excitation in the transmitting coil as well as receiving coil. The characteristics of the magnetic field vary with the relative position of the transmitting coil and the receiving coil. Therefore, the standard database of magnetic flux density under different offsets is established. During receiver positioning, the magnetic sensor array collects the magnetic flux density data, which is fused later. The position of the receiving coil can be detected by comparing it with the standard data.

**Figure 5:**
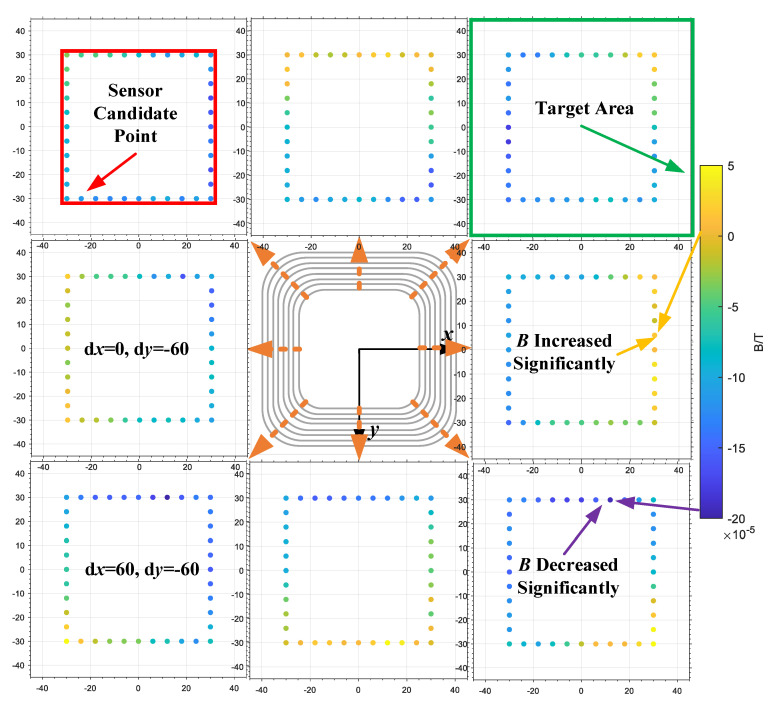
Magnetic field variation law of candidate position under different receiving coil displacement directions.

Due to the limitation of cost, space and complexity of the matching circuit, the sensor applied to sample the magnetic field characteristics cannot cover the whole object area. Combined with geometric characteristics of the square coil, a series of candidate sensor positions are selected. Then, observe the characteristics of these points under different relative displacements of the magnetic coupler. The number and geometric distribution of sensors are ultimately determined to provide a basis for positioning algorithm design. The magnetic flux density of the receiving coil is simulated shifted 60 mm along the eight directions indicated by the arrow in Figure 5. The obtained magnetic flux density values of each point in the object area are subtracted from that of each point in the positive case as shown in Figure 5. The magnetic field change of the sensors’ candidate position in the object area indicates that when the receiving coil is offset in a certain direction, the magnetic flux density at the candidate point at the far end of this direction shows the largest change trend. The offset direction of the receiving coil can be judged preliminarily.

Employing a cubic spline curve to fit different offset and magnetic flux density curves, a standard database of magnetic induction strength of receiving coil under different offset is established to position the receiving coil precisely. Set the cubic spline function’s expression to

(13) $$s(x)=a_{0}x^{3}+a_{1}x^{2}+a_{2}x^{1}+a_{3}x^{0}$$

**Figure 6:**
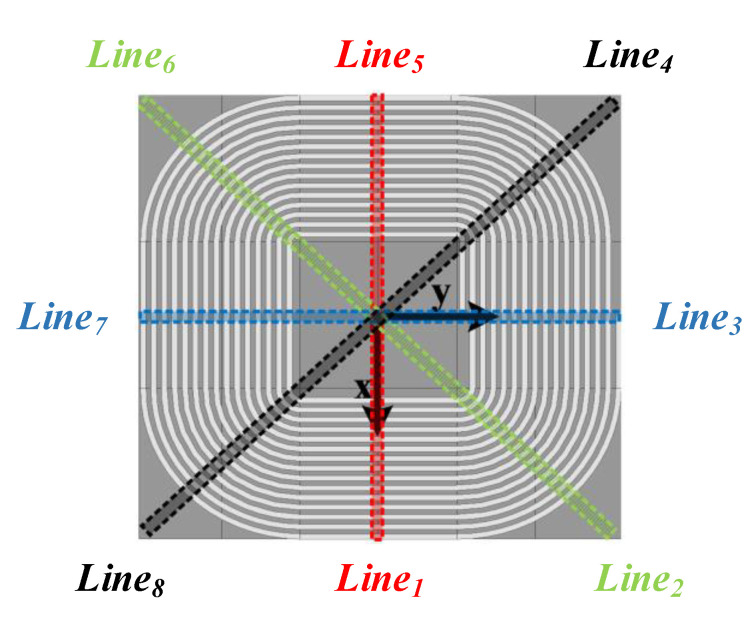
Diagram of typical offset direction.

In the cubic spline curve, the curve segment between two nodes is fitted by a cubic polynomial and the whole spline curve is composed of several cubic polynomial curves that include their own coefficients. Curve fitting with cubic spline function can still have high accuracy with fewer sampling points. As shown in Figure 6, eight typical directions are selected symmetrically on the surface of the transmitting coil to fit the magnetic flux density curves with different offsets in these eight typical directions.

Table 3 and Table 4 show the relationship between the offset in different directions and the magnetic flux density as the sampling points for the cubic spline curve fitting.

**Figure 7:**
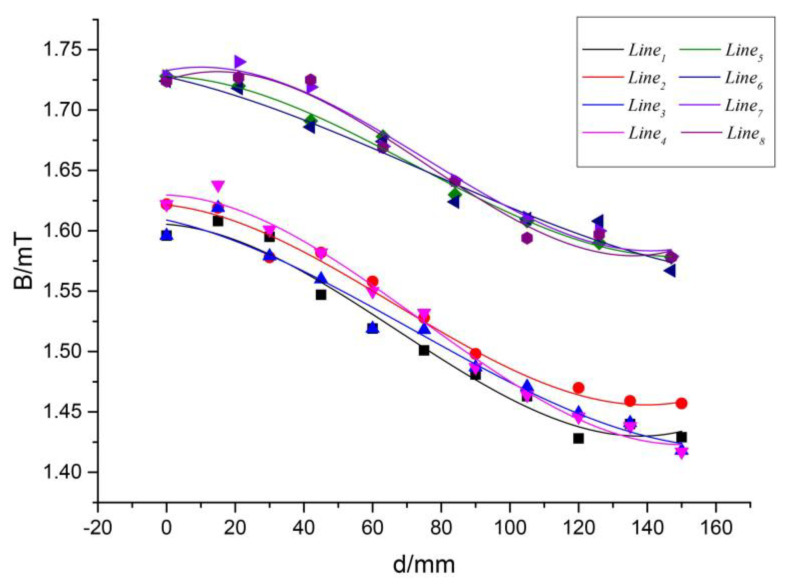
Fitting data of magnetic flux density in eight typical directions.

Fitting curves with cubic splines based on the data in Table 3 and Table 4 are shown in Figure 7. Residuals of the curve fitting between offset and magnetic induction strength in all eight directions are less than 0.03. The real-time accurate positioning of the receiving coil can be achieved by comparing the fitted curve data with the sensor array detection data.

**Figure 8:**
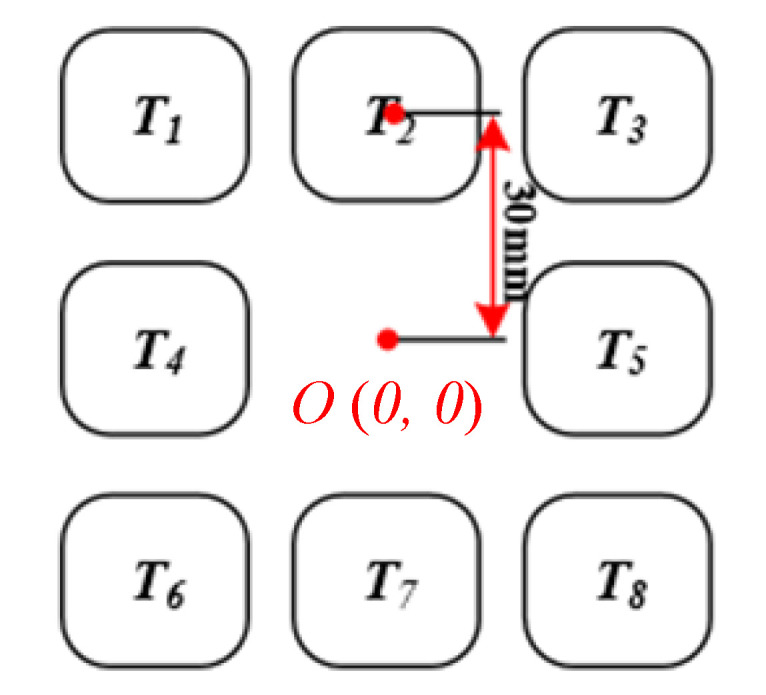
Diagram of sensors layout.

The arrangement of the Planar 8-direction symmetric magnetic sensor is shown in Figure 8. When the transmitting coil and the receiving coil are aligned, the output value *T_i0_* of each sensor is stored. The detection installation is switched on when the receiving coil approaches the transmitting coil. Read the measured value *T_i_* continuously from the sensor array, then according to (14), calculate the difference between the current measured value and the prepared value in the case of the positive alignment.

(14) $$\Delta T_{i}=T_{i}-T_{i0}(i=1,2\ldots \ldots 7,8)$$

Firstly, the direction of the receiving coil offset according to (15).

(15) $$i=\arg\max D_{i}$$

In the light of the calculated *i* value, the migration direction can be preliminarily determined, corresponding to the direction from Line_1_ to Line_8_ in Figure 5. Using the sensor layout proposed in Figure 6, *D_i_* is calculated according to (16).

(16) $$\{\begin{array}{c} D_{1}=\Delta T_{6}+\Delta T_{7}+\Delta T_{8}-\Delta T_{1}-\Delta T_{2}-\Delta T_{3}\\ D_{2}=\Delta T_{5}+\Delta T_{7}+\Delta T_{8}-\Delta T_{1}-\Delta T_{2}-\Delta T_{4}\\ D_{3}=\Delta T_{3}+\Delta T_{5}+\Delta T_{8}-\Delta T_{1}-\Delta T_{4}-\Delta T_{6}\\ D_{4}=\Delta T_{2}+\Delta T_{3}+\Delta T_{5}-\Delta T_{4}-\Delta T_{6}-\Delta T_{7}\\ D_{5}=\Delta T_{1}+\Delta T_{2}+\Delta T_{3}-\Delta T_{6}-\Delta T_{7}-\Delta T_{8}\\ D_{6}=\Delta T_{1}+\Delta T_{2}+\Delta T_{4}-\Delta T_{5}-\Delta T_{7}-\Delta T_{8}\\ D_{7}=\Delta T_{1}+\Delta T_{4}+\Delta T_{6}-\Delta T_{3}-\Delta T_{5}-\Delta T_{8}\\ D_{8}=\Delta T_{4}+\Delta T_{6}+\Delta T_{7}-\Delta T_{2}-\Delta T_{3}-\Delta T_{5} \end{array}$$

Then, according to (17), calculate the magnetic flux density of the offset in different directions from Line_1_ to Line_8_.

(17) $$\{\begin{array}{c} B=\frac{1}{2}(T_{4}+T_{5})i=1,5\\ B=\frac{1}{2}(T_{3}+T_{6})i=2,6\\ B=\frac{1}{2}(T_{2}+T_{7})i=3,7\\ B=\frac{1}{2}(T_{1}+T_{8})i=4,8 \end{array}$$

Location information is obtained by comparing the obtained with the magnetic flux density values at different locations shown in Figure 7 to complete the positioning of the receiving coil.

### 3.3. Constant Current Control Strategy

For square coils, the mutual inductance varies with the relative position of the coil, with the maximum mutual inductance in the case of alignment. Changes in the relative positions of the transmitting and receiving coils alter the magnetic field in space for two reasons.

First, the change of the magnetic field caused by the change of the relative position of the coils. Second, the change of the magnetic field resulting from the change of current intensity in the coil, which is caused by the change of mutual inductance. In the methods of detection and positioning of magnetic fields, it is essential to distinguish the magnetic field changes from these two factors. Keeping the current of the primary and secondary sides constant, the factors leading to the magnetic field change due to the mutual inductance change disappear, which reduces the variables for the magnetic field detection and improves the detection accuracy.

For a WPT system with an S–S topology, the primary current and the secondary current are calculated as follows

(18) $$I_{s}=\frac{V_{inv}}{\omega M}$$

(19) $$I_{p}=\frac{I_{s}R_{eq}}{\omega M}$$

where *I_s_* is the secondary current, *V_inv_* is the output voltage of the primary side inversion, *I_p_* is the primary current and *R_eq_* is the equivalent resistance of the secondary side. *ω* is the resonance frequency and *M* is the mutual inductance.

**Figure 9:**
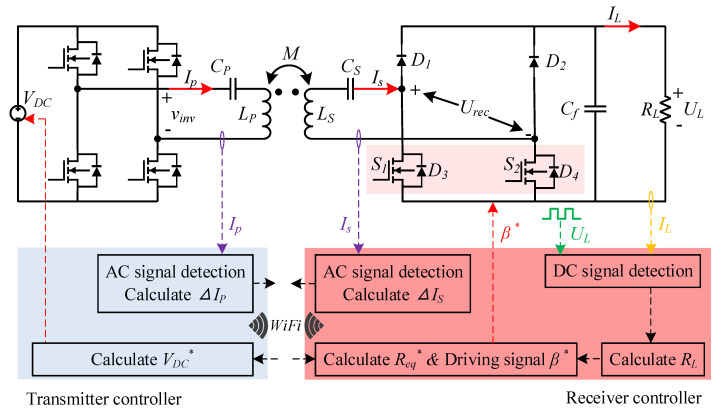
Schematic diagram of constant current control.

The schematic diagram of constant current control of the primary and secondary sides is shown in Figure 9. V_DC_^*^ is the expected direct-current input voltage of the transmitter, Δ*I_P_* and Δ*I_s_* are the difference between the measured value of primary and secondary current and the expected value, R_eq_^*^ is the reflection impedance and β^*^ is the conduction angle of the controllable rectifier. Table 5 is used to describe the symbols in Figure 9.

The primary and secondary current of coil alignment is taken as the preset standard current value. Firstly, the secondary current *I_s_* is collected by the AC Hall sensor. Δ*I_s_* is obtained by calculating the difference between the measured value and the preset value. It is sent to the transmitter controller with the WiFi module. The coil misalignment will lead to the change of mutual inductance. When the resonant frequency is constant, Equation (18) shows that the secondary side constant current can be achieved by adjusting the output voltage of the inverter. The DC input voltage of a full-bridge inverter is proportional to the output voltage. The driving signal V_DC_^*^ is calculated by Δ*I_s_*, and the constant current of the primary side is realized by adjusting *V_DC_* with a simple circuit structure. Similarly, Δ*I_p_* is calculated and sent to the receiver controller. Δ*I_p_* is used as the basis for the receiver controller to calculate the expected equivalent load impedance R_eq_^*^. By adjusting the conduction angle β^*^ of the controllable rectifier, the equivalent load impedance can be adjusted to the desired value, and the primary current *I_P_* is constant.

**Figure 10:**
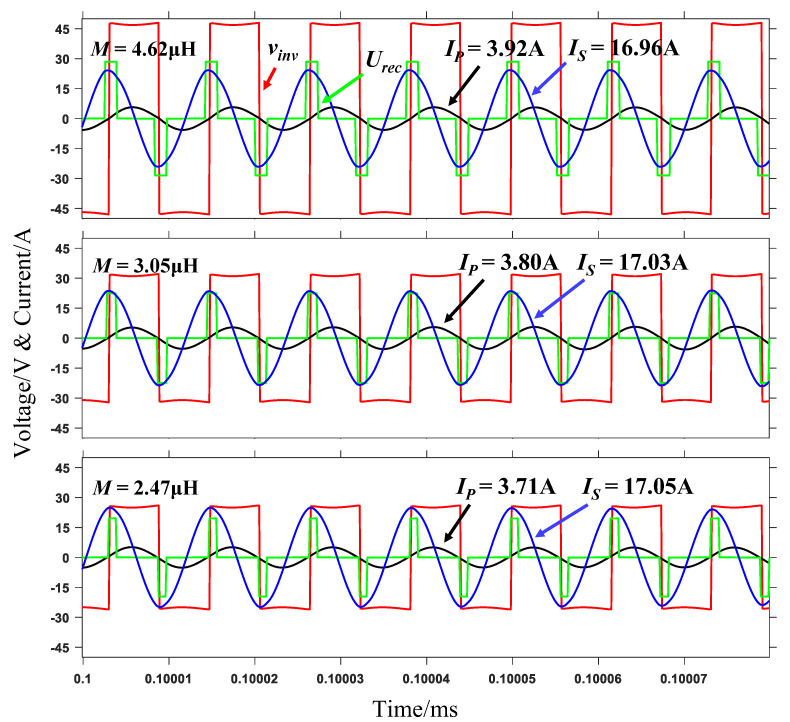
Working waveforms in different offset states.

A reference power of 150 W is provided when the magnetic coupler is directly facing, and set the current reference value at this time. Constant current control under different mutual inductances when the offset is 0 (positive alignment), 75 and 90 mm is implemented. The steady-state working waveform of the three cases is shown in Figure 10. As the offset value increases, the mutual inductance decreases, the output voltage of the inverter and input voltage of the controllable rectifier decreases, the conduction angle of the controllable rectifier decreases gradually, but the current remains relatively constant, reaching the control target.

## 4. Experimental Verification

### 4.1. Experimental Setup

**Figure 11:**
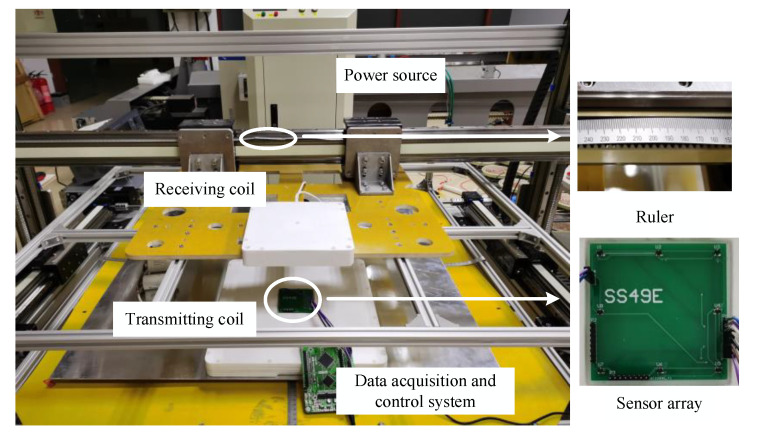
Experimental platform for coil positioning.

**Figure 12:**
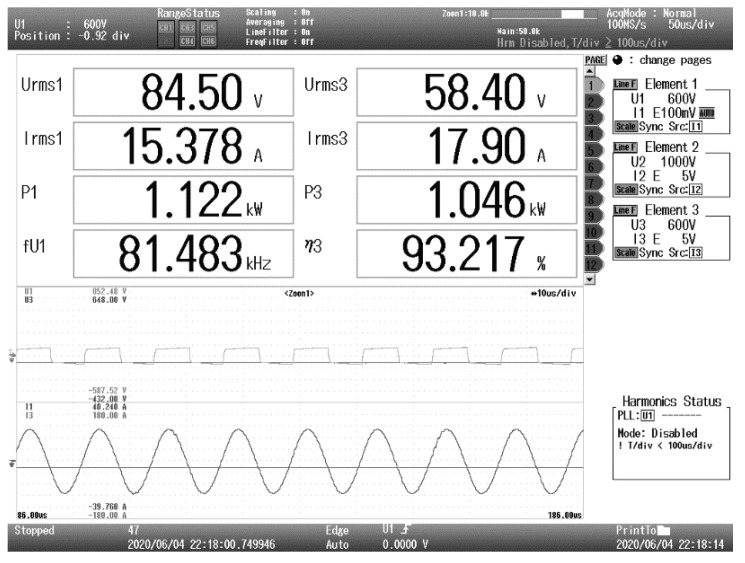
Working parameters of the coil positioning system.

As shown in Figure 11, a magnetic coupler prototype of a 1 kW AGV WPT system is built to verify the coil positioning method. The efficiency of the WPT system is 93.2%, as shown in Figure 12. The outer diameters of the square transmitting coil and receiving coil are 300 and 150 mm, respectively. The coil positioning experiment was carried out under the low-power excitation of 150 W. The magnetic flux density is less than 1.8 mT in the entire wireless charging area for the safety of the electromagnetic environment. A linear Hall sensor SS49E is used to measure the magnetic flux density, which has the advantages of microstructure, magnetic optimization packaging and low noise output. The measurement range of SS49E is ±1500 Gs, which conforms to the space magnetic flux density range of magnetic coupler under low-power excitation. The resolution of SS49E is 2.5 mV/Gs, which can realize high-precision linear detection. The sensor array is integrated on a 75 × 75 mm circuit board and placed at the center of the transmitter. The data acquisition system with STM32F103RCT6 microprocessor as the core collects and adjusts the sensor signals, and runs the positioning algorithm.

### 4.2. Magnetic Alignment Evaluation

**Figure 13:**
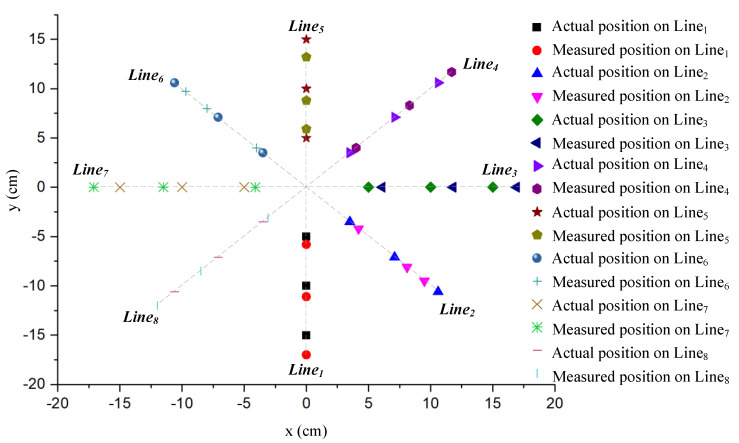
Comparison between the measured value and the actual value in each direction.

To verify the feasibility and accuracy of the proposed coil positioning method, taking the center of the transmitter as the origin point, the receiving coil is tested for positioning in increments of 5 cm each time in eight typical directions as shown in Figure 6, with an offset range of 0–15 cm. The actual positions and the measured positions at each test point are shown in Figure 13.

**Figure 14:**
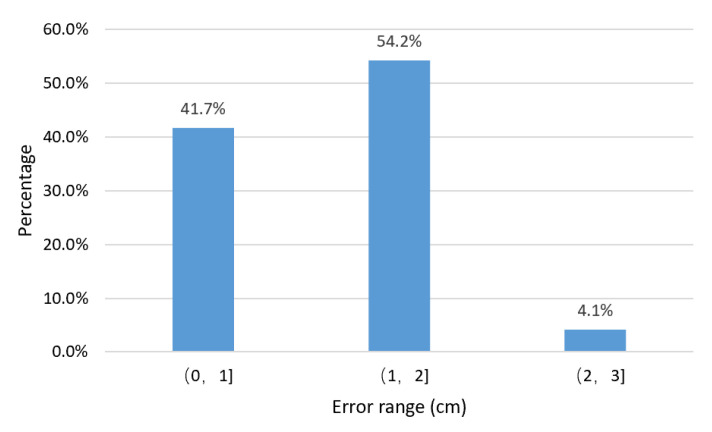
Positioning error distribution.

Figure 14 shows the proportion of the positioning error distribution of the 24 test samples. The percentage of error represents the proportion of the number of experimental samples in the total number of experimental samples distributed within the error range. According to Figure 14, 41.7% of the 24 samples have a measurement error of less than 1 cm, 54.2% have an error of between 1 and 2 cm and 4.1% are from 2 to 3 cm. This shows that 95.9% of the sample errors are not more than 2 cm for a misalignment range from −15 to 15 cm.

**Figure 15:**
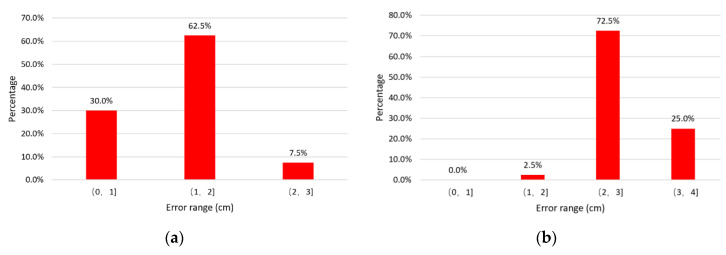
Positioning error distribution: (**a**) with the offset range of 1–10 cm; (**b**) with the offset range of 16–25 cm.

Regulate the offset range to 1–10 cm and 16–25 cm, respectively, and adjust the offset increment to 1 cm. Take the directions of Line1, Line_3_, Line_5_ and Line_7_ in Figure 6 for instance to test the position of the receiving coil more precisely. The probability distribution of positioning error is shown in Figure 15.

The results show that the small offset is beneficial to coil positioning, which has higher positioning accuracy. As a consequence of the stronger magnetic field at this time, the sensor generates a higher sensor output. Larger sensor output can increase the resolution over offset, leading to a higher positioning accuracy in the case of smaller offset.

**Figure 16:**
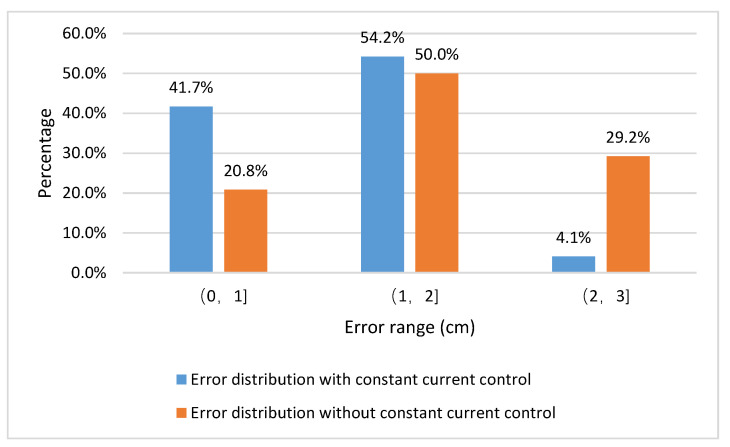
Comparison of positioning error distribution.

When the resonator constant current strategy fails in the system, taking the center of the transmitter as the origin point, the receiving coil is tested for positioning in increments of 5 cm each time in eight typical directions as shown in Figure 6, with an offset range of 0–15 cm. Positioning error distribution with constant current and without constant current is shown in Figure 16. The proportion of test samples distributed in the error range of 0–1 cm decreased, and the proportion of test samples distributed in the error range of 2–3 cm increased. It can be found that the constant current control can improve the accuracy of coil positioning, which has an important role.

In summary, among 104 samples tested by the method presented in this paper, 21.2% of the sample positioning errors are between 0 and 1 cm, 37.5% of the sample positioning errors are between 1 and 2 cm, 31.7% are between 2 and 3 cm and 9.6% are between 3 and 4 cm. This indicates that when the receiving coil offset range is from −25 to +25 cm, there are more than 90.4% of the samples whose error does not exceed 3 cm.

## 5. Conclusions

This paper presents a high-precision AGV WPT system receiving the coil positioning method based on the magnetic field measurement and multisensor data fusion. The magnetic field distribution of alignment and misalignment between transmitting and receiving coils is analyzed. Based on the difference of magnetic field distribution in the above two cases, the magnetic flux density component of the measured point in the vertical direction can provide the misalignment information of the coil on the xoy plane. The magnetic field changes of coils with different offsets are simulated, and it is proved that the magnetic induction intensity changes most in the direction of the offset. Then, the offset direction of the receiving coil can be determined. The magnetic flux density data measured by the sensor array is fused and compared with the standard database of the magnetic flux density under different offset conditions of the receiver coil, and the receiver coil can be accurately positioned. A double-sided resonator constant current strategy is proposed, which eliminates the interference factor of the magnetic field distribution affected by mutual inductance changes and improves the positioning accuracy. Finally, a 1 kW AGV wireless charging platform is built. The outer diameter of the transmitter and receiver is 300 and 150 mm, respectively. Among 104 test samples, the maximum positioning error of the proposed receiving coil positioning method is less than 4 cm within the offset range from −25 to +25 cm. The positioning error of 90.4% samples is less than 3 cm.

## Figures and Tables

**Table 1:** Comparison of existing positioning methods.

Method	Accuracy	Cost	Complexity	Advantage	Disadvantage
GPS [17,18]	2–10 m	Low	Medium	Universality	Low robustness and low accuracy
Camera [19]	1.2–2.8 cm	High	Medium	High accuracy	Environment dependency
RFID [20,21]	2.5–6 cm	Medium	Medium	High accuracy	Complex deployment Environment dependency
UWB [22]	10–50 cm	High	High	High resolution	High cost
Magnetic Coupling [10,23,24,25]	1–10 cm	Low	Low	Magnetic interference	High robustness and high accuracy

**Table 2:** Design parameters of the magnetic coupler.

Parameter	Transmitter Coil	Receiver Coil
External diameter	300 mm	150 mm
Turns	15	9
Wire spacing	3 mm	2.4 mm
Wire radius	3.6 mm	3.6 mm
Self-inductance	89.68 μH	12.16 μH
Mutual inductance	4.62 μH	
Coupling coefficient	0.14	

**Table 3:** Magnetic flux density B with different misalignment (Line_1_, Line_3_, Line_5_, Line_7_).

Misalignment/mm	B (Line_1_)/mT	B (Line_3_)/mT	B (Line_5_)/mT	B (Line_7_)/mT
0	1.596	1.622	1.596	1.622
15	1.608	1.619	1.619	1.638
30	1.595	1.578	1.579	1.601
45	1.547	1.582	1.56	1.582
60	1.519	1.558	1.519	1.550
75	1.501	1.528	1.518	1.532
90	1.481	1.498	1.487	1.487
105	1.463	1.468	1.471	1.464
120	1.428	1.470	1.449	1.446
135	1.440	1.459	1.441	1.438
150	1.429	1.457	1.418	1.417

**Table 4:** Magnetic flux density B with different misalignment (Line_2_, Line_4_, Line_6_, Line_8_).

Misalignment/mm	B (Line_2_)/mT	B (Line_4_)/mT	B (Line_6_)/mT	B (Line_8_)/mT
0	1.728	1.724	1.728	1.724
21	1.720	1.718	1.740	1.727
42	1.691	1.686	1.719	1.725
63	1.678	1.674	1.670	1.669
84	1.630	1.624	1.642	1.641
105	1.608	1.610	1.611	1.594
126	1.590	1.608	1.600	1.597
147	1.578	1.567	1.579	1.578

**Table 5:** Symbol explanation in Figure 9.

Symbol	Expression	Value
*V_DC_*	Actual direct-current input voltage	48 V
*I_p_*	Primary current	3.9 A
*I_s_*	Secondary current	16.9 A
*M*	Mutual inductance	4.62 μH
*R_L_*	Equivalent resistance of the load	2.4 Ω
*C_P_*	Primary compensation capacitor	39.14 nF
*C_S_*	Secondary compensation capacitor	288.55 nF
*C_f_*	Filter capacitor	470 μF
*L_P_*	Primary self-induced	89.68 μH
*L_S_*	Secondary self-induced	12.16 μH
